# Significance of bile culture surveillance for postoperative management of pancreatoduodenectomy

**DOI:** 10.1186/s12957-019-1773-7

**Published:** 2019-12-30

**Authors:** Keishi Sugimachi, Tomohiro Iguchi, Yohei Mano, Masaru Morita, Masaki Mori, Yasushi Toh

**Affiliations:** 1 Department of Hepatobiliary-Pancreatic Surgery, National Hospital Organization Kyushu Cancer Center, 3-1-1 Notame, Minami-ku, Fukuoka, 811-1395 Japan; 2 Department of Gastroenterological Surgery, National Hospital Organization Kyushu Cancer Center, 3-1-1 Notame, Minami-ku, Fukuoka, 811-1395 Japan; 3 Department of Surgery and Science, Graduate School of Medical Sciences, Kyushu University, 3-1-1 Maidashi, Higashi-ku, Fukuoka, 812-8582 Japan

**Keywords:** Bile culture, Pancreatoduodenectomy, Surgical site infection, Multi-drug resistance

## Abstract

**Background:**

The management of infectious complications is important in pancreatoduodenectomy (PD). We sought to determine the significance of preoperative surveillance bile culture in perioperative management of PD.

**Methods:**

This study enrolled 69 patients who underwent PD for malignant tumors at a single institute between 2014 and 2017. Surveillance bile culture was performed before or during surgery. Correlations between the incidence of infectious postoperative complications and clinicopathological parameters, including bile cultures, were evaluated.

**Results:**

Preoperative positive bile culture was confirmed in 28 of 51 patients (55%). Bile culture was positive in 27 of 30 cases (90%) with preoperative biliary drainage, and 1 of 21 cases (5%) without drainage (*p* < 0.01). Preoperative isolated microorganisms in bile were consistent with those detected in surgical sites in 11 of 27 cases (41%). Cases with positive multi-drug-resistant bacteria in preoperative bile culture showed significantly higher incisional SSI after PD (*p* = 0.01). The risk factors for the incidence of organ/space SSI were soft pancreatic texture (*p* = 0.01) and smoking history (*p* = 0.02) by multivariate analysis. Preoperative positive bile culture was neither associated with organ/space SSI nor overall postoperative complications.

**Conclusions:**

Preoperative surveillance bile culture is useful for the management of wound infection, prediction of causative pathogens for infectious complications, and the selection of perioperative antibiotic prophylaxis.

## Background

Pancreatoduodenectomy (PD) is the only curative option for pancreatic, biliary, and duodenal malignant tumors. Perioperative management of patients undergoing PD has been dramatically improved in past few decades, but the morbidity still ranges from 20 to 50% [1, 2]. The complication and death rates of PD mainly depend on major infectious complications, including postoperative pancreatic fistula (POPF) and biliary leakage, and many other host factors, such as infection, the severity of jaundice, nutritional status, and impaired organ functions [3, 4].

Obstructive jaundice is the most common symptom for periampullary and ampullary tumors that need PD; thus, biliary drainage has been commonly performed. Preoperative biliary drainage re-establishes entero-hepatic circulation, provides symptomatic relief to patients with pruritus and cholangitis, and serves as a temporizing measure if surgery is delayed or neoadjuvant therapy is considered [5]. However, controversy remains about the pre-operative management of obstructive jaundice undergoing planned PD, because bacterobilia induced by biliary drainage has been reported to be associated with postoperative infectious complication and also even triggers the development of POPF [6–9]. Several studies reported the importance of specific prophylaxes based on bile culture to prevent postoperative complications, but preoperative antibiotics could potentially induce colonization of multi-drug-resistant (MDR) pathogens in the biliary tract [10, 11]. Though both claims related to biliary drainage may be logically correct, it is important to care and strictly manage biliary infection before PD.

To establish effective and safe strategies for prophylaxis of perioperative infectious complication, more accurate information on the incidence of perioperative biliary infection in patients who have undergone PD is needed. Therefore, we conducted a retrospective study of surveillance bile culture to analyze recent trends and significance of biliary infection on the perioperative outcome of PD.

## Methods

### Patients

A retrospective cohort study was performed with 69 consecutive patients who underwent PD at a single institute (Kyushu Cancer Center) between the years 2014 and 2017. Data from all these patients were collected from medical charts and analyzed. The demographic data of patients are summarized in Table 1.

**Table 1:** Demographics and clinical characteristics of the study population

Factor		Number
Sex	Male/female	45/24
Age	years	67.3 ± 9.9
Disease	Pancreas tumor/bile duct tumor/duodenal tumor	40/22/7
Smoking history	Current (never or ever)	17 (24.6%)
Body mass index		22.3 ± 2.9
Diabetes mellitus		22 (31.9%)
Preoperative biliary stent placement		34 (49.3%)
Surgical procedure	PD/PPPD/SSPPD	11/8/50
Episode of preoperative cholangitis		13 (18.9%)

Abbreviations: *PD* pancreatoduodenectomy, *PPPD* pylorus preserving pancreatoduodenectomy, *SSPPD* subtotal stomach preserving pancreatoduodenectomy

### Perioperative management

Preoperative biliary drainage was performed in 34 of 69 patients (49%). Endoscopic retrograde (internal) biliary drainage was done in 30 cases (43%), endoscopic nasobiliary drainage was done in 2 cases (2%), and percutaneous transhepatic biliary drainage was done in 2 cases (2%). Patients treated with antimicrobial agents for high fever or abnormal liver function tests were diagnosed as cholangitis. As a surveillance bile culture, bile specimens were collected at the time of either preoperative biliary drainage procedure or surgery. Multi-drug-resistant pathogens were defined as described elsewhere [12, 13].

The child method was applied for the reconstruction of PD. An internal or external stent was placed at pancreatojejunostomy, and the abdominal cavity was routinely drained using closed suction tubes in all patients. Second-generation cephalosporins were used as prophylactic antibiotics for all patients at least 2 days after operation without regard to the preoperative bile culture.

All infectious complications were proven by a positive culture of purulent discharge. POPF was defined according to the International Study Group of Pancreatic Fistula [14], and the grade of complication was defined by Clavien-Dindo classification [15].

### Statistical analysis

All statistical analyses were performed using JMP 13.0 software (SAS; Cary, NC, USA). All variables were expressed as the mean ± standard deviation. Continuous variables were compared using the Mann–Whitney *U* test. Categorical data were compared using the *χ*^2^ test. Logistic regression analysis was performed to identify the independent predictors of complications. A *p* value of < 0.05 was regarded as statistically significant.

## Results

### Pre- and intra-operative surveillance bile culture

Preoperative and intraoperative bile culture was performed in 12 (17%) and 46 patients (67%), respectively. Both pre- and intra-operative bile culture were done in 7 patients; thus, surveillance bile culture was done in 51 patients (74%) overall. Bile culture was positive in 28 of 51 patients (55%), and MDR pathogens were detected in 11 of 51 patients (22%). In 7 patients, those both pre- and intra-operative bile culture were done, the bile culture was positive at preoperative drainage in 4 cases and became positive after drainage (intraoperatively) in 3 cases. In 3 of 4 cases, the MDR pathogens matched between preoperative and intraoperative cultures. Detected MDR pathogens were *Enterobacter* species in 7, methicillin-resistant *Staphylococcus aureus* in 2, *Pseudomonas* species in 1, and extended spectrum β-lactamase-producing strains in 1 case. Bile culture was positive in 27 of 30 cases (90%) with preoperative biliary drainage, and 1 of 21 cases (5%) without drainage with statistically significant difference (*p* < 0.01). All 11 patients those MDR pathogens were detected had undergone biliary drainage. MDR pathogens were detected by preoperative culture in 2 patients, intraoperative culture in 8 patients, and both in 1 patient.

### Postoperative complications

Relationship between surveillance bile culture and postoperative complications was analyzed in 51 cases those surveillance bile culture was done (Table 2). Incisional and organ/space surgical site infection (SSI) occurred in 6 (12%) and 33 (65%) cases, respectively. The occurrence of incisional SSI was significantly higher in the cases with MDR pathogen positive in bile culture (*p* = 0.01). There was no significant correlation between the morbidity of organ/space SSI and surveillance bile culture.

**Table 2:** Postoperative complications and surveillance bile culture

Complication	All cases	Bile culture positive	*p* value	MDR pathogens positive	*p* value
	(*n* = 51)	(*n* = 28)		(*n* = 11)	
Incisional SSI (%)	6 (12)	4 (14)	0.53	4 (36)	0.01
Organ/space SSI (%)	33 (65)	18 (64)	0.95	7 (63)	0.93
Bile leakage (%)	4 (8)	2 (7)	0.84	1 (9)	0.86
Pancreatic fistula (%)	21 (41)	12 (43)	0.79	4 (36)	0.71
Cholangitis (%)	3 (6)	2 (7)	0.67	1 (9)	0.63
Enteritis (%)	3 (6)	2 (7)	0.67	1 (9)	0.63
Clavien-Dindo ≥ 3 complication (%)	16 (31)	7 (25)	0.28	3 (27)	0.74

Abbreviations: *SSI* surgical site infection, *MDR* multi-drug resistance

### Coincidence of pre-operative bile culture and post-operative abdominal fluid culture

Bile cultures were postoperatively obtained only in 7 cases because external biliary drainage was not applied in most cases. In the postoperative course, the culture of abdominal fluid from intra-abdominal drains was obtained from 54 of the 69 patients. The results of preoperative bile culture and postoperative abdominal fluid culture were compared. The postoperative culture was positive in 37 cases (68%), and MDR pathogens were positive in 23 cases (43%). Both pre- and post-operative cultures were obtained in 48 cases. The coincidence of pre- and post-operative culture was examined in 26 cases, and those preoperative bile cultures were positive. The coincidence of pathogens was observed in 11 cases (42%) (Table 3), and 10 out of these 11 cases, the detected bacteria were causative pathogens of SSI. In 10 cases of MDR pathogen testing positive in preoperative bile culture, 8 (80%) showed the same pathogens in abdominal fluid postoperatively, and the rate of coincidence of detected pathogens was significantly higher in cases with MDR-positive compared to non MDR-positive (*p* < 0.01, Table 3). Detected MDR pathogens were *Enterobacter* species in 5, methicillin-resistant *Staphylococcus aureus* in 2, and *Pseudomonas* species in 1 case.

**Table 3:** Coincidence of pathogens in preoperative and postoperative culture

Culture	Coincidence of pathogens	Non-coincidence of pathogens	*p* value
	(*n* = 11)	(*n* = 15)	
Drug sensitive pathogen positive (*n* = 16)	3 (19%)	13 (81%)	< 0.01
Multi-drug resistant pathogen positive (*n* = 10)	8 (80%)	2 (20%)	

### Risk factors associated with morbidity of organ/space SSI

Risk factors associated with morbidity of organ/space SSI were analyzed. In univariate analysis, diagnosis of pancreatic adenocarcinoma, operative time ≥ 360 min, blood loss ≥ 400 g, soft pancreatic texture, and main pancreatic duct diameter ≤ 3 mm were the risk factors of occurrence of organ/space SSI (Table 4). In multivariate analysis, smoking history and pancreatic texture were the independent risk factors associated with organ/space SSI (Table 4). The preoperative bile culture was not statistically associated with the occurrence of organ/space SSI.

**Table 4:** Risk factors associated with morbidity of organ/space surgical site infection

Factors		Univariate analysis			Multivariate analysis	
		oSSI(−) *n* = 26	oSSI(+) *n* = 43	*p* value	hazard ratio (95% CI)	*p* value
Diagnosis	PDAC/others	20/6	20/23	0.01		
Smoking history	(+)/(−)	4/22	13/30	0.16	32.7 (1.65–647)	0.02
Operative time (min)	≥ 360/≤ 359	13/13	32/11	0.04		
Blood loss (g)	≥ 400/≤ 399	16/10	14/29	0.02		
Pancreatic texture	Soft/hard	5/21	31/11	< 0.01	17.2 (1.77–168)	0.01
Main pancreatic duct diameter (mm)	≤ 3/≥ 4	9/17	26/13	0.01		
Preoperative bile culture	Positive/negative	10/16	18/25	0.78		
Drug resistant pathogen in preoperative bile culture	Positive/negative	4/22	7/36	0.92		

Abbreviations: *PDAC* pancreatic adenocarcinoma, *oSSI* organ/space surgical site infection, *CI* confident interval

## Discussion

We have shown that bacterobilia is induced by preoperative biliary obstruction and drainage and associated with higher incidence of incisional SSI. The coincidence of pathogens, especially MDR, between bile and post-operative infectious lesions, such as intraabdominal fluid, were frequently observed in our series. These results indicated that colonization in bile due to pre-operative biliary drainage could be causative pathogens of postoperative infectious complications.

Postoperative pancreatic fistula is a major determinant of postoperative infectious complications, and it is associated with mortality of PD. Thus, the management of infectious complications is critical in perioperative care [16, 17]. Our multivariate analysis indicated that bacterobilia is not an independent risk factor for organ/space SSI, but pancreatic texture and smoking history were associated with the incidence of SSI. Those results were also observed by the analysis of 51 cases those surveillance bile culture was done (data not shown). Soft pancreatic parenchyma and small pancreatic duct diameter are a well-established risk factor of POPF [18, 19], and our present data also indicated that risk factors for POPF are dominant compared with bacterobilia in the incidence of organ/space SSI.

The present data, consistent with the literature, showed that preoperative biliary drainage caused bacterobilia [6]. Benefits of preoperative biliary drainage for patients undergoing PD remain largely controversial [7, 20–22]. Several studies question its value given morbidity and complications associated with biliary infection. However, decreased jaundice can relieve patient symptom and improve immunonutritious conditions through entero-hepatic circulation [23]. More importantly, neoadjuvant preoperative chemotherapy is becoming more common for borderline resectable pancreatic adenocarcinoma; thus, preoperative biliary drainage is essential for at least certain patient groups [24]. Our study indicated that the most serious complications mainly depend on the risk factors of POPF, but not on bacterobilia; thus, biliary drainage is acceptable if necessary. In cases with biliary drainage, it is important to apply strict management to prevent infectious post-operative complications. We recommend the use of specific antibiotics based on surveillance bile culture to avoid colonization of MDR bacteria and to manage surgical incisions as contaminated wounds. Our study supports the theory reported by Okamura et al. that preoperative bile culture-target prophylactic antibiotics decreased SSI following hepato-pancreato-biliary surgery [25].

We acknowledge limitations of this non-randomized study, including relatively small sample size. Many studies reported that bile is contaminated in patients undergoing PD after biliary drainage [6–10, 20, 21]. Our study agrees with the importance of surveillance bile culture for management of patients with PD. The present study described the impact of bacterobilia caused by MDR pathogens, which is becoming more important with the current spread of antibiotic-resistant bacteria, not only in bile but in postoperative infectious lesions.

## Conclusions

Higher incidence of incisional SSI is anticipated in patients undergoing PD with biliary drainage due to bacterobilia. Management of wounds as contaminated and use of appropriate prophylactic antimicrobial agents based on surveillance bile culture are needed to improve operative outcomes.

## Data Availability

All data analyzed during this study are included in this published article.

## Abbreviations

MDR: Multi-drug resistant
PD: Pancreatoduodenectomy
POPF: Postoperative pancreatic fistula
SSI: Surgical site infection
